# Contralateral dominance emerges from geometric transformation in bilateral control systems

**DOI:** 10.3389/fncom.2026.1839583

**Published:** 2026-04-29

**Authors:** Nobuchika Yamaki, Tenna Churiki

**Affiliations:** 1 TNQ Tech, Co., Newark, DE, United States; 2 King’s College London, London, United Kingdom

**Keywords:** axial twist, bilateral control, computational neuroanatomy, contralateral organization, decussation

## Abstract

**Introduction:**

Contralateral organization is a defining feature of vertebrate nervous systems, yet its functional origin remains incompletely understood. We examined whether contralateral routing can arise as an advantageous solution in delayed bilateral control systems using a minimal computational framework.

**Methods:**

We constructed abstract bilateral sensorimotor networks composed of sensory, central, and motor units on the left and right sides, and systematically compared alternative architectures differing in sensory laterality, commissural coupling, and local connectivity. We evaluated one-dimensional and two-dimensional models, introducing in the latter a continuous twist parameter representing transformations between sensory and motor coordinate relationships. Dense parameter scanning and bootstrap analysis were used to estimate the transition point and its robustness.

**Results:**

In one-dimensional models, contralateral configurations were dynamically viable but sensitive to the choice of objective function. In two-dimensional models, the twist parameter reorganized the architecture landscape: without transformation, optimal solutions were predominantly ipsilateral, whereas under strong transformation they became predominantly contralateral. Intermediate conditions exhibited an abrupt transition rather than a gradual shift. Dense parameter scanning localized this transition to a threshold at θ_c ≈ 0.483. Bootstrap analysis showed that this threshold was stable (95% CI: 0.481766–0.483507) and only weakly dependent on longitudinal delay. Objective values were minimized near and just above the transition region.

**Discussion:**

These results indicate that, within an abstract dynamical framework, contralateral routing can become advantageous under conditions of transformed sensorimotor relationships and delayed interactions.

## 1 Introduction

Contralateral organization is a fundamental feature of vertebrate nervous systems, whereby sensory inputs and motor outputs are predominantly represented in the opposite hemisphere. This organization is evident across multiple levels of neural structure, including the decussation of retinal projections at the optic chiasm, contralateral motor control mediated by corticospinal pathways, and interhemispheric coordination via commissural systems such as the corpus callosum (Jeffery, 2001; Herrera and García-Frigola, 2008; Gazzaniga, 2000). Despite its ubiquity, the functional basis of contralateral organization remains incompletely understood.

Several explanatory frameworks have been proposed. Developmental and evolutionary accounts suggest that contralateral wiring arises from large-scale transformations of body plan geometry, including hypotheses such as axial twist or inversion during early embryogenesis (de Lussanet and Osse, 2012; Chédotal, 2019). Other approaches emphasize wiring economy and network efficiency, proposing that neural systems minimize connection length or metabolic cost while maintaining functional integration (Cherniak, 1994; Bullmore and Sporns, 2012). In addition, studies of spinal and cortical systems highlight the role of commissural pathways in coordinating bilateral activity and enabling integrated behavior (Kiehn, 2016; Maxwell et al., 2020; Roland et al., 2017). Classical neurobiological perspectives have also emphasized the importance of preserving orderly spatial relations in sensory representations, particularly in visual systems in which inversion of the sensory image must be reconciled with coherent mapping of external space (Cajal, 1995; Jeffery, 2001; Kaas, 1997). While these perspectives provide important insights, they primarily address how contralateral organization may arise, be maintained, or be developmentally constrained, rather than under what functional conditions contralateral routing becomes advantageous relative to ipsilateral alternatives.

This issue is especially important because contralateral organization is not uniform across all sensory and motor systems. Some modalities exhibit strongly structured cross-midline organization, whereas others do not. More generally, the functional consequences of laterality depend on how sensory coordinates, internal processing, and motor outputs are related to one another. Systems that preserve continuous spatial mappings may be subject to different architectural pressures from systems such as olfaction, which is less organized by a continuous topographic representation of external space and is correspondingly more ipsilateral in its primary projections (Shepherd, 2004). From a systems-level perspective, the problem can therefore be reframed not simply as one of anatomical laterality, but as one of architecture selection under dynamical and dynamical constraints and transformation structure.

Neural systems operate under delayed signal propagation, noise, and the need for stable bilateral coordination. In such systems, the relationship between sensory inputs and motor outputs depends not only on anatomical connectivity but also on the effective transformation between sensory and motor coordinate systems. When this relationship is direct, ipsilateral routing may be sufficient. When it is inverted or mismatched, however, different routing architectures may become functionally preferable. Thus, changes in the geometry of sensorimotor mapping may bias which bilateral architecture best supports accurate and robust behavior.

Recent work in computational neuroscience has emphasized how network architecture shapes dynamical function, including the effects of delays, recurrent interactions, and interhemispheric coupling on stability and coordination (Deco et al., 2011; Breakspear, 2017). However, most existing models assume a fixed relationship between sensory and motor coordinates and do not consider how transformations of this relationship influence the selection of bilateral architectures. As a result, it remains unclear whether contralateral routing can become favored as a consequence of general dynamical constraints, rather than being imposed *a priori* or attributed only to specific developmental histories.

Here, we address this question using a minimal computational framework that isolates the interaction between coordinate transformation, delay, and bilateral competition. We model a bilateral sensorimotor system composed of sensory, integrative, and motor components in each hemisphere, and systematically compare alternative architectures that differ in sensory laterality and interhemispheric coupling. To represent transformations of sensorimotor geometry, we introduce a continuous twist parameter that denotes the degree of inversion or mismatch between sensory and motor coordinate systems, that is, the extent to which spatial relations in sensory coordinates must be transformed to support appropriate motor mapping.

Our central hypothesis is that increasing sensorimotor transformation can shift the functional preference of the system toward cross-midline routing when delayed signal propagation and bilateral competition are present. Specifically, we test whether increasing transformation produces a sharp reorganization in the optimal architecture, such that ipsilateral mappings dominate under low transformation and contralateral mappings dominate under high transformation. Importantly, contralateral routing is not hard-wired as the outcome of the model, but is evaluated as one candidate solution within an abstract bilateral control system.

By combining exhaustive architecture comparison with dense parameter exploration, this study aims to identify the conditions under which contralateral routing becomes functionally advantageous in a minimal delayed dynamical system. In this sense, the present work does not attempt to reproduce the full anatomical or developmental complexity of vertebrate nervous systems. Rather, it seeks to identify a minimal dynamical principle by which transformations in sensorimotor geometry can bias the selection of bilateral routing architectures, thereby providing an abstract theoretical bridge between classical geometric accounts and systems-level dynamical considerations.

## 2 Materials and methods

### 2.1 Overview of the modeling framework

**FIGURE 1:**
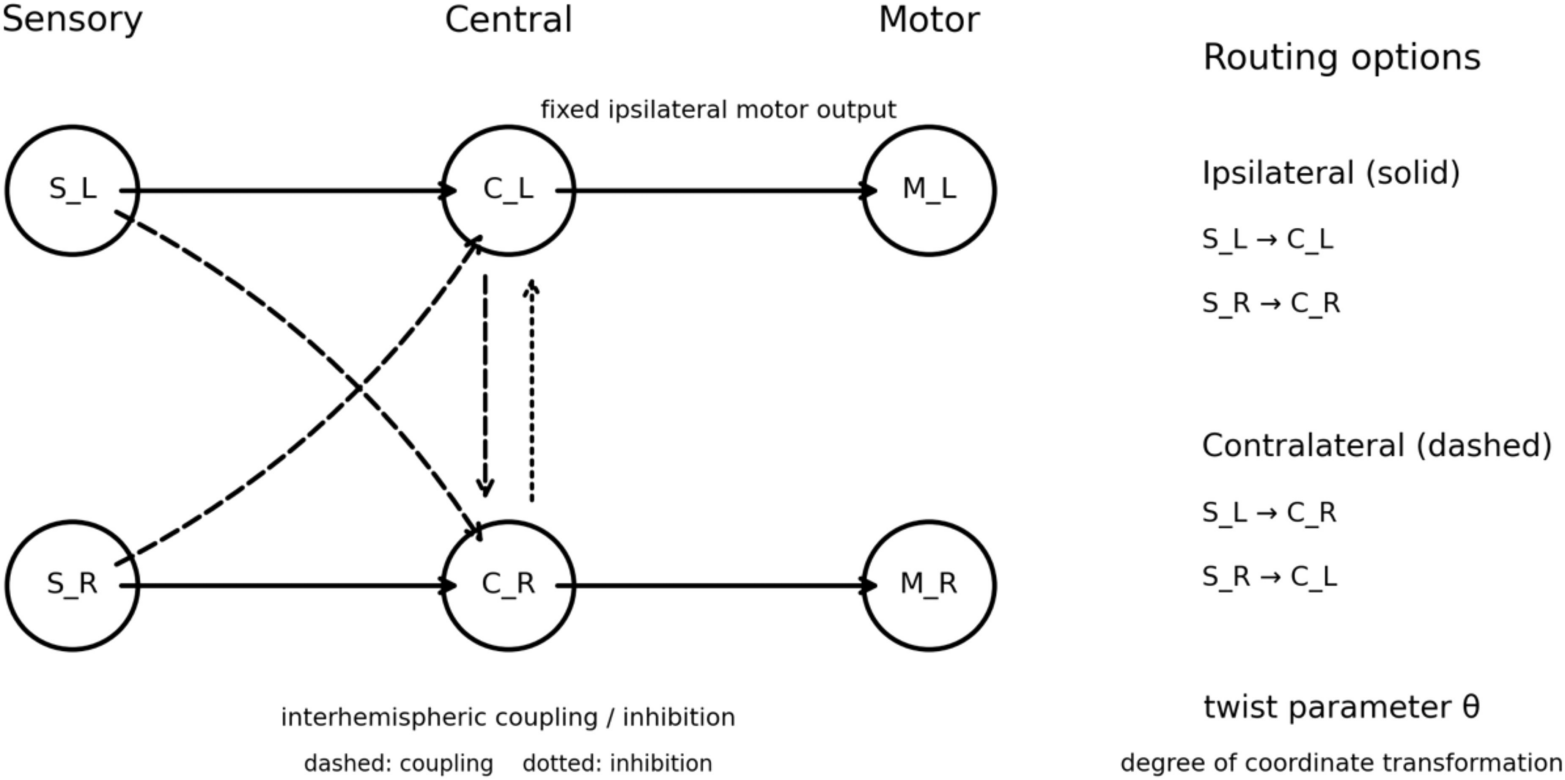
Minimal bilateral sensorimotor model with alternative sensory routing architectures. The model consists of left and right sensory (S_L, S_R), central (C_L, C_R), and motor (M_L, M_R) units. Sensory inputs can be routed either ipsilaterally (solid lines) or contralaterally (dashed lines), while motor output is fixed as ipsilateral. Central units may interact via interhemispheric coupling and inhibition. The twist parameter θ represents the degree of transformation between sensory and motor coordinate systems, ranging from near-direct correspondence at low θ to increasingly mismatched or inverted relationships at high θ. The model is intentionally abstract and does not represent anatomical structures directly, but provides a minimal framework for comparing routing architectures under controlled dynamical conditions.

We constructed a minimal bilateral sensorimotor network to examine how contralateral organization can emerge from the interaction among delayed signal propagation, bilateral competition, and transformed sensorimotor mapping (Figure 1). The model consisted of two homologous pathways, left and right, each containing a sensory unit, a central integrative unit, and a motor unit. These pathways were treated as abstract bilateral processing channels rather than as anatomically explicit hemispheres with defined spatial embedding, cell types, or developmental identities. The purpose of the model was therefore not to reproduce vertebrate neuroanatomy directly, but to isolate the dynamical conditions under which alternative bilateral routing architectures become advantageous in a delayed control system.

At each point in parameter space, all candidate architectures were evaluated under identical task, noise, and delay conditions. Architecture selection was defined by the objective value of each candidate network, allowing the preferred routing pattern to be identified without hard-wiring contralateral organization as the outcome.

### 2.2 Network architecture

The model comprised six nodes:


S⁢_⁢L,S⁢_⁢R

sensory nodes


C⁢_⁢L,C⁢_⁢R

central integrative nodes


M⁢_⁢L,M⁢_⁢R

motor nodes

where the subscripts *L* and *R* denote the left and right pathways. Three architectural factors were varied. First, sensory input could be routed either ipsilaterally or contralaterally. In the ipsilateral configuration, *S_L* projected to *C_L* and *S_R* projected to *C_R*. In the contralateral configuration, *S_L* projected to *C_R* and *S_R* projected to *C_L*. Second, excitatory commissural coupling between the two central nodes could be either present or absent. Third, local sensory-central coupling within each side could be either present or absent. When present, this local coupling was bidirectional, linking *S_L* with *C_L* and *S_R* with *C_R*. These three binary factors generated *2 × 2 × 2 = 8* candidate architectures.

Motor output was fixed as ipsilateral in all simulations:


C⁢_⁢L→M⁢_⁢L

C⁢_⁢R→M⁢_⁢R

This restriction reduced the architectural degrees of freedom and allowed the analysis to focus specifically on sensory laterality and interhemispheric central interactions.

### 2.3 Connection weights, delays, and inhibitory competition

Connection strengths were fixed across simulations. Sensory-to-central projections had weight *w_SC = 1.0*. Local sensory-central feedback connections had weight *w_CS = 0.4*. Central-to-motor projections had weight *w_CM = 1.0*. Excitatory commissural connections had weight *w_CC = 0.7*. The gain of the nonlinear activation function was set to *g = 1.0*.

Two delay scales were used. A short delay of


d⁢_⁢short=1

time step was assigned to sensory-central projections, local within-side sensory-central feedback connections, central-to-motor projections, and inhibitory interhemispheric coupling. A longer delay,


d⁢_⁢l⁢o⁢n⁢g∈{1,2,…,20},

was assigned only to excitatory commissural coupling between *C_L* and *C_R* when that coupling was present. Thus, *d_long* selectively controlled the delay of long-range excitatory interhemispheric communication.

Interhemispheric competition was implemented as reciprocal inhibition between the two central nodes. The inhibitory weight was defined as


w⁢_⁢i⁢n⁢h=−I⁢_⁢i⁢n⁢h⁢i⁢b*w⁢_⁢i⁢n⁢h⁢i⁢b⁢_⁢s⁢c⁢a⁢l⁢e

where


w⁢_⁢i⁢n⁢h⁢i⁢b⁢_⁢s⁢c⁢a⁢l⁢e=1.0

and


I⁢_⁢i⁢n⁢h⁢i⁢b∈{0.0,0.2,0.5,0.8,1.2,1.8}.

Accordingly, when inhibitory coupling was present, the model included


C⁢_⁢L←C⁢_⁢R⁢with⁢weight⁢w⁢_⁢i⁢n⁢h⁢and⁢delay⁢d⁢_⁢s⁢h⁢o⁢r⁢t

C⁢_⁢R←C⁢_⁢L⁢with⁢weight⁢w⁢_⁢i⁢n⁢h⁢and⁢delay⁢d⁢_⁢s⁢h⁢o⁢r⁢t

### 2.4 Node dynamics

At each time step, the state of node *i* was updated according to a nonlinear rate-based rule:


x⁢_⁢i⁢(t+1)=tanh⁢(g*net⁢_⁢i⁢(t))

where *x_i(t)* is the state of node *i*, *g* is the gain parameter, and *net_i(t)* is the total input to that node at time *t*. The total input was defined as the sum of delayed synaptic inputs, externally applied task input, and additive Gaussian noise:


n⁢e⁢t⁢_⁢i⁢(t)=s⁢u⁢m⁢_⁢j⁢[w⁢_⁢i⁢j*x⁢_⁢j⁢(t−d⁢_⁢i⁢j)]+I⁢_⁢i⁢(t)+epsilon⁢_⁢i⁢(t)

where *w_ij* denotes the connection weight from node *j* to node *i*, *d_ij* is the corresponding transmission delay, *I_i(t)* denotes externally applied task input to the sensory nodes, and *epsilon_i(t)* is zero-mean Gaussian noise. At each time step, external task input was applied only to the sensory nodes. Specifically, *u_L(t)* and *u_R(t)* were added to *S_L* and *S_R*, respectively, whereas no direct external input was applied to the central or motor nodes.

This formulation provided a bounded nonlinear integration rule while preserving a minimal dynamical structure.

### 2.5 Sensorimotor task

The network performed a continuous tracking task. In the one-dimensional simulations, the system tracked a target position along the lateral axis. In the two-dimensional simulations, the system tracked target positions along both the lateral (*x*) and orthogonal (*y*) axes. Target amplitudes were fixed at


x⁢_⁢t⁢a⁢r⁢g⁢e⁢t⁢_⁢a⁢m⁢p=1.0

y⁢_⁢t⁢a⁢r⁢g⁢e⁢t⁢_⁢a⁢m⁢p=1.0

At each time step, the sign of the target could switch stochastically. Along the lateral axis, sign reversal occurred with probability


p⁢_⁢s⁢w⁢i⁢t⁢c⁢h=0.08

and, in the two-dimensional case, independent sign reversal along the orthogonal axis occurred with probability


p⁢_⁢s⁢w⁢i⁢t⁢c⁢h⁢_⁢y=0.08.

Tracking errors were defined as


e⁢_⁢x⁢(t)=x⁢_⁢target⁢(t)−x⁢_⁢body⁢(t)

and, in the two-dimensional case,


e⁢_⁢y⁢(t)=y⁢_⁢target⁢(t)−y⁢_⁢body⁢(t)

where *x_body(t)* and *y_body(t)* denote the current body position along the two task dimensions. Body position was updated using the motor output of the network. Along the lateral axis, the left and right motor nodes acted in opposition:


x⁢_⁢body⁢(t+1)=x⁢_⁢body⁢(t)+k⁢_⁢move*(m⁢_⁢R⁢(t)−m⁢_⁢L⁢(t))

+eta⁢_⁢x⁢(t)

where *m_L(t)* and *m_R(t)* are the activities of *M_L* and *M_R*, respectively,


k⁢_⁢m⁢o⁢v⁢e=0.55,

and *eta_x(t)* is Gaussian process noise with standard deviation


p⁢r⁢o⁢c⁢_⁢n⁢o⁢i⁢s⁢e⁢_⁢x=0.02.

In the two-dimensional simulations, the orthogonal axis was controlled by a fixed proportional feedback rule rather than by the bilateral network itself:


y⁢_⁢body⁢(t+ 1)=y⁢_⁢body⁢(t)+k⁢_⁢y*e⁢_⁢y⁢(t)+eta⁢_⁢y⁢(t)

where


k⁢_⁢y=0.55

and *eta_y(t)* is Gaussian process noise with standard deviation


p⁢r⁢o⁢c⁢_⁢n⁢o⁢i⁢s⁢e⁢_⁢y=0.02.

This arrangement allowed lateralized architecture selection to be evaluated primarily through the *x* dimension, while the *y* dimension served as a stabilizing task component in the two-dimensional setting.

## 2.6 Transformation parameter

To represent transformations in sensorimotor mapping, we introduced a continuous parameter *theta*, constrained to the interval


0≤theta≤=1.

In the present model, *theta* did not represent a literal anatomical twist. Rather, it denoted the degree of inversion or mismatch between the sensory error structure and the motor routing required to correct that error. The limiting cases were defined as follows:


t⁢h⁢e⁢t⁢a=0

purely ipsilateral mapping


t⁢h⁢e⁢t⁢a=1

fully inverted mapping

Intermediate values of *theta* were implemented as the simplest continuous interpolation between these two limiting mappings.

For the lateral tracking error *e_x(t)*, ipsilateral and contralateral sensory drive components were defined using half-wave rectification:


ipsi⁢_⁢L⁢(t)=max⁢(−e⁢_⁢x⁢(t), 0)

i⁢p⁢s⁢i⁢_⁢R⁢(t)=m⁢a⁢x⁢(+e⁢_⁢x⁢(t), 0)

contra⁢_⁢L⁢(t)=max⁢(+e⁢_⁢x⁢(t), 0)

contra⁢_⁢R⁢(t)=max⁢(−e⁢_⁢x⁢(t), 0)

The actual sensory inputs to the left and right sensory nodes were then given by


u⁢_⁢L⁢(t)=( 1−theta)*ipsi⁢_⁢L⁢(t)+theta*contra⁢_⁢L⁢(t)

u⁢_⁢R⁢(t)=( 1−theta)*ipsi⁢_⁢R⁢(t)+theta*contra⁢_⁢R⁢(t)

These task-dependent inputs were added to *S_L* and *S_R* at each time step. Thus, small values of *theta* favored near-direct ipsilateral correspondence between sensory error and motor correction, whereas large values progressively favored inverted correspondence.

### 2.7 Objective function

Architecture performance was quantified using a multi-component objective function:


J=lambda⁢_⁢track*J⁢_⁢track+lambda⁢_⁢effort*J⁢_⁢effort+lambda

_⁢a⁢s⁢y⁢m*J⁢_⁢asym+lambda⁢_⁢delay*J⁢_⁢delay+lambda⁢_⁢noise

*J⁢_⁢noise

where *J_track* is tracking cost, *J_effort* is motor effort cost, *J_asym* is asymmetry cost, *J_delay* is delay-related cost, and *J_noise* is noise-sensitivity cost. Baseline weighting coefficients were set to


l⁢a⁢m⁢b⁢d⁢a⁢_⁢t⁢r⁢a⁢c⁢k=1.0

l⁢a⁢m⁢b⁢d⁢a⁢_⁢e⁢f⁢f⁢o⁢r⁢t=0.08

l⁢a⁢m⁢b⁢d⁢a⁢_⁢a⁢s⁢y⁢m=0.05

l⁢a⁢m⁢b⁢d⁢a⁢_⁢d⁢e⁢l⁢a⁢y=1.0

l⁢a⁢m⁢b⁢d⁢a⁢_⁢n⁢o⁢i⁢s⁢e=1.0

Tracking cost was defined as the time-averaged squared tracking error. In the one-dimensional simulations,


J⁢_⁢track=mean⁢_⁢t⁢[e⁢_⁢x⁢(t)⁢∧⁢2]

whereas in the two-dimensional simulations,


J⁢_⁢track=mean⁢_⁢t⁢[e⁢_⁢x⁢(t)⁢∧⁢2+e⁢_⁢y⁢(t)⁢∧⁢2]

Motor effort cost was defined as the time-averaged summed squared motor activity:


J⁢_⁢effort=mean⁢_⁢t⁢[m⁢_⁢L⁢(t)⁢∧⁢2+m⁢_⁢R⁢(t)⁢∧⁢2]

Asymmetry cost was defined as the time-averaged squared difference between left and right motor activity:

J_*asym*=*mean*_t[(m_L(t)−m_R(t))∧2]Delay-related cost was computed from the mean absolute activity of each source node, weighted by the absolute connection strength and transmission delay:


J⁢_⁢d⁢e⁢l⁢a⁢y=s⁢u⁢m⁢_⁢(i,j)⁢[d⁢_⁢i⁢j*|w⁢_⁢i⁢j|*m⁢e⁢a⁢n⁢_⁢t⁢(|x⁢_⁢j⁢(t)|)]

This term penalized architectures that relied more strongly on delayed communication. Noise-sensitivity cost was defined as the additional tracking cost observed when external noise amplitude was increased by a multiplicative factor *kappa*:


J⁢_⁢noise=J⁢_⁢track⁢_⁢high⁢_⁢noise−J⁢_⁢track⁢_⁢baseline

with


k⁢a⁢p⁢p⁢a=1.6

To compute this term, each architecture was evaluated both under the baseline noise level *sigma* and under an elevated noise level *kappa × sigma*. The objective function was therefore used as a common operational criterion for comparing candidate architectures rather than as a unique definition of biological optimality.

To assess the dependence of the results on the weighting scheme, we additionally performed a one-factor-at-a-time sensitivity analysis. In that analysis, each weighting coefficient was independently scaled to *0.5×*, *1.0×*, and *1.5×* of its baseline value while the remaining coefficients were held fixed. The resulting effects on the estimated transition point and on contralateral dominance in the high-transformation regime were then quantified.

### 2.8 Simulation protocol

All simulations were run for


T=220

time steps. The first


w⁢a⁢r⁢m⁢u⁢p=20

time steps were excluded from analysis, and all summary measures were computed over the remaining post-warmup period. At each point in parameter space, all candidate architectures were evaluated under identical conditions, and the optimal architecture was defined as the one with the lowest objective value *J*.

To reduce stochastic dependence on a single realization, repeated simulations were performed for each architecture and parameter combination. In the exhaustive one-dimensional scan, each condition was evaluated with 12 repetitions. In the inhibitory one-dimensional scan, the tracking-cost one-dimensional scan, the one-dimensional endpoint comparison, the two-dimensional baseline scan, the discrete two-dimensional twist scan, and the dense two-dimensional twist scan, each condition was evaluated with 10 repetitions. In the pairwise comparison and architecture-class comparison analyses, repeated single-run evaluations were performed across five initial random realizations for each condition. Independent random number generators were initialized with distinct seeds for each repetition.

The primary outcome was the proportion of optimal solutions exhibiting contralateral sensory routing, denoted


c⁢r⁢o⁢s⁢s⁢_⁢f⁢r⁢e⁢q.

At each point in parameter space, this quantity took the value 1 when the best-performing architecture used contralateral sensory routing and 0 otherwise. Averaging across cells or conditions therefore yielded the frequency with which contralateral architectures were selected as optimal.

### 2.9 Parameter exploration

The model was evaluated across multiple parameter grids. The long excitatory commissural delay was varied over


d⁢_⁢l⁢o⁢n⁢g⁢i⁢n⁢{1,2,…,20}.

Noise amplitude was varied over the set


sigmain{0.01,0.02,0.03,0.04,0.05,0.055,0.06,0.065,

0.07,0.08,0.09,0.10,0.11,0.12}

for the main inhibitory and two-dimensional analyses, and over a closely related set including 0.045 but excluding 0.09 and 0.11 in selected one-dimensional scans. Interhemispheric inhibitory strength was varied over


I⁢_⁢i⁢n⁢h⁢i⁢b⁢i⁢n⁢{0.0,0.2,0.5,0.8,1.2,1.8}.

For the two-dimensional transformation analyses, discrete twist conditions of


t⁢h⁢e⁢t⁢a⁢i⁢n⁢{0,1/3,2/3,1}

were first evaluated, followed by a dense scan over


thetain{0.25,0.30,0.35,0.40,0.45,0.50,0.55,0.60,0.65,

0.70,0.75,0.80,0.85}.

This dense sampling was used to localize the transition between ipsilateral-dominated and contralateral-dominated regimes.

### 2.10 Definition of the transition point

The transition point, denoted *theta_c*, was defined as the value of *theta* at which


c⁢r⁢o⁢s⁢s⁢_⁢f⁢r⁢e⁢q=0.5.

For a given cross-frequency curve sampled at discrete twist values, *theta_c* was estimated by linear interpolation between the two neighboring values of *theta* that bracketed the 0.5 crossing. When the crossing occurred between consecutive points *theta_k* and *theta_(k+1)* with corresponding values *f_k* and *f_(k+1)*, the estimate was computed as


theta_c=theta_k+(0.5−f_k)/(f_(k+1)−f_k)*

(t⁢h⁢e⁢t⁢a⁢_⁢(k+1)−t⁢h⁢e⁢t⁢a⁢_⁢k)

provided that *f_k* and *f_(k+1)* lay on opposite sides of 0.5. This interpolation procedure was applied both to the global dense twist scan and to delay-specific cross-frequency curves.

### 2.11 Bootstrap analysis

The robustness of the transition was evaluated by bootstrap resampling of parameter-space points from the dense two-dimensional twist scan. In each bootstrap replicate, the full set of simulated cells was resampled with replacement, preserving the original sample size. A new mean *cross_freq* versus *theta* curve was then computed from the resampled data, and the corresponding *theta_c* value was re-estimated using the same interpolation rule described above. This yielded a bootstrap distribution of global transition points.

To assess the dependence of the transition on excitatory commissural delay, the same procedure was repeated separately for each value of *d_long*. For each replicate, a delay-specific estimate *theta_c(d_long)* was obtained, and a linear slope relating *theta_c* to *d_long* was computed across the full delay range. The bootstrap analysis used


B=2000

resamples, from which median values and 95% confidence intervals were obtained for the global transition point and for the slope of its delay dependence.

### 2.12 Mechanistic analysis

To examine the mechanism underlying architecture selection, we quantified temporal mismatch and sign alignment between the required lateral motor correction and the motor command produced by the network. The required lateral correction was given by the instantaneous tracking error *e_x(t)*, whereas the produced lateral motor command was given by the difference between right and left motor activity:


u⁢_⁢x⁢(t)=m⁢_⁢R⁢(t)−m⁢_⁢L⁢(t)

Temporal mismatch was defined as the absolute difference between these two quantities:


mismatch⁢(t)=|e⁢_⁢x⁢(t)−u⁢_⁢x⁢(t)|

Sign alignment was defined as an indicator of whether the motor command had the correct sign relative to the required correction:


a⁢l⁢i⁢g⁢n⁢m⁢e⁢n⁢t⁢(t)=1,i⁢f⁢s⁢i⁢g⁢n⁢(e⁢_⁢x⁢(t))=s⁢i⁢g⁢n⁢(u⁢_⁢x⁢(t))

alignment(t) = 0, otherwise

For each architecture and twist condition, these measures were averaged across post-warmup time steps and then summarized across repetitions. This analysis was used to determine whether the twist-dependent shift in optimal architecture was associated with systematic changes in the temporal consistency between task demands and motor output.

## 3 Results

### 3.1 Contralateral architectures are not globally optimal in one-dimensional systems

We first examined whether contralateral organization is favored in a minimal one-dimensional setting. In pairwise comparisons across 260 parameter-space points, the proportion of conditions in which contralateral architectures outperformed ipsilateral ones was 0.503, with a mean objective difference of 0.00021. Mean objective values were 2.172 for contralateral architectures and 2.171 for ipsilateral architectures.

Exhaustive architecture selection across all candidate configurations yielded a mean contralateral frequency (cross_freq) of 0.490, with values ranging from 0.083 to 0.833.

These results indicate that contralateral architectures are dynamically viable but do not dominate the tested one-dimensional solution space.

### 3.2 Objective structure strongly modulates architecture selection

We next examined how the definition of the objective function influences architecture selection. Under an inhibitory scan across 1,680 parameter-space points, the mean contralateral frequency was 0.504, with values ranging from 0 to 0.900.

When the objective function emphasized tracking performance, contralateral architectures were strongly suppressed. Under this formulation, the mean contralateral frequency decreased to 1.19 × 10^4^, with a maximum of 0.100. The mean objective value increased to 11.181. Mean cost components were 7.363 (tracking), 6.615 (high-noise tracking), 0.535 (effort), 0.853 (asymmetry), 4.481 (delay), and −0.748 (noise sensitivity).

These results show that architecture selection in the present framework depends strongly on the balance between competing cost components.

### 3.3 Geometric transformation reorganizes the architecture landscape

We next introduced a geometric transformation of sensorimotor coordinates using the twist parameter θ. In the two-dimensional baseline condition without inversion (θ = 0), the system remained overwhelmingly ipsilateral, with a mean contralateral frequency of 0.000179 across 1,680 parameter-space points.

Under full inversion (θ = 1), contralateral architectures dominated completely, with a mean contralateral frequency of 1.000. Mean objective values decreased from 11.181 at θ = 0 to 7.144 at θ = 1, and mean tracking cost decreased from 7.363 to 3.306.

These results indicate that geometric transformation reorganizes the solution landscape such that ipsilateral-dominated regimes at low twist are replaced by contralateral-dominated regimes at high twist within the present abstract control framework.

### 3.4 A sharp transition in optimal architecture occurs with increasing twist

To characterize this reorganization in detail, we evaluated both discrete and densely sampled values of the twist parameter.

In discrete conditions, the mean contralateral frequency was 0.000238 at *theta* = 0, 0.0863 at *theta* = 1/3, 0.999881 at *theta* = 2/3, and 1.000 at *theta* = 1.

**FIGURE 2:**
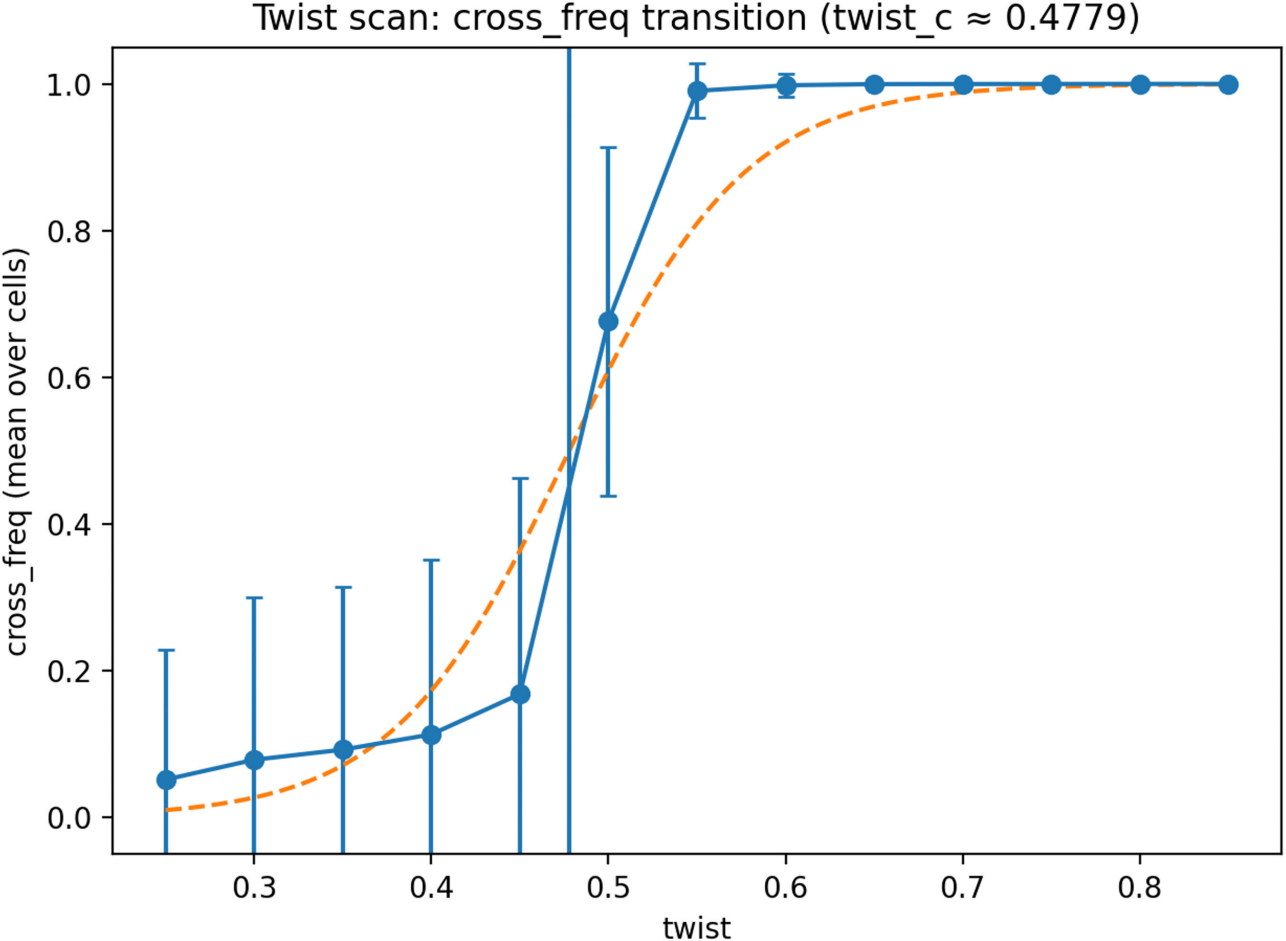
Twist-dependent shift in contralateral dominance. Mean contralateral frequency (cross_freq) as a function of the geometric twist parameter θ in the dense two-dimensional twist scan. Contralateral dominance remained low at small θ values, increased sharply between θ = 0.45 and 0.50, and saturated near 1 at larger θ values. The dashed curve shows a fitted sigmoid function for visualization, and the vertical line indicates the estimated transition point (θ_c ≈ 0.483). These results show that the shift from ipsilateral to contralateral dominance occurs over a narrow range of θ, rather than as a gradual linear change.

A dense scan of the twist parameter further localized this transition (Figure 2). Mean contralateral frequencies were 0.0519 at *theta* = 0.25, 0.0789 at *theta* = 0.30, 0.0927 at *theta* = 0.35, 0.113 at *theta* = 0.40, 0.168 at *theta* = 0.45, 0.677 at *theta* = 0.50, 0.991 at *theta* = 0.55, 0.998 at *theta* = 0.60, 0.9997 at *theta* = 0.65, and 0.9999 at *theta* = 0.70. At higher values (*theta* = 0.75, 0.80, 0.85), contralateral dominance remained saturated near 1.

The transition point, defined as the value of *theta* at which *cross_freq* reached 0.5 by linear interpolation between neighboring sampled values, was estimated as *theta_c* = 0.482623.

### 3.5 The transition is weakly dependent on interhemispheric delay

**FIGURE 3:**
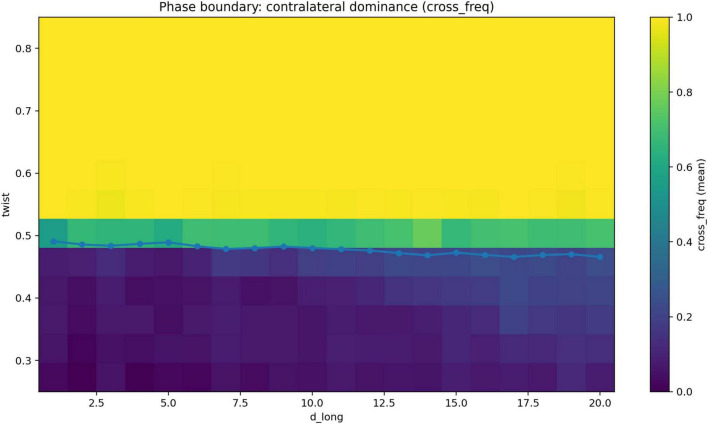
Architecture boundary in the (θ, d_long) parameter space. Two-dimensional map of contralateral frequency (cross_freq) across the parameter space defined by the geometric twist parameter θ and longitudinal delay (d_long). Warmer values indicate stronger contralateral dominance, whereas cooler values indicate ipsilateral dominance. The overlaid boundary indicates the estimated transition between the two regimes. The boundary is approximately horizontal across the full range of d_long, indicating that the transition is primarily governed by θ and only weakly influenced by longitudinal delay.

We next examined the dependence of the transition on interhemispheric delay. The two-dimensional phase diagram (Figure 3) shows that the boundary separating ipsilateral and contralateral regimes is nearly horizontal across the full range of d_long.

**FIGURE 4:**
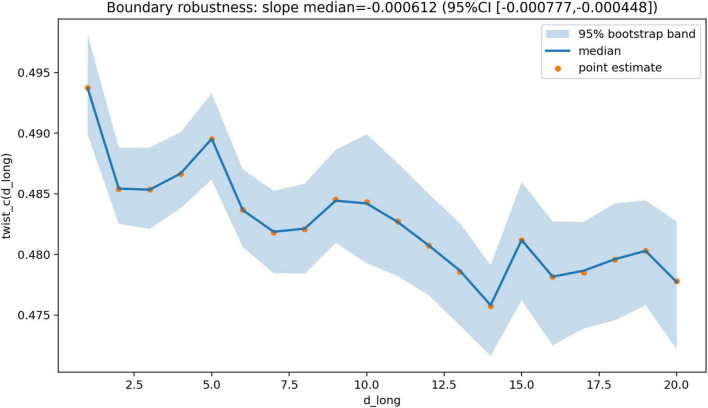
Delay dependence of the transition point. Estimated transition point θ_c(d_long) plotted as a function of longitudinal delay (d_long), with bootstrap confidence band. Points indicate point estimates, the solid line indicates the median bootstrap estimate, and the shaded region indicates the 95% bootstrap interval. The shallow slope indicates that the transition point depends only minimally on longitudinal delay across the tested range.

**FIGURE 5:**
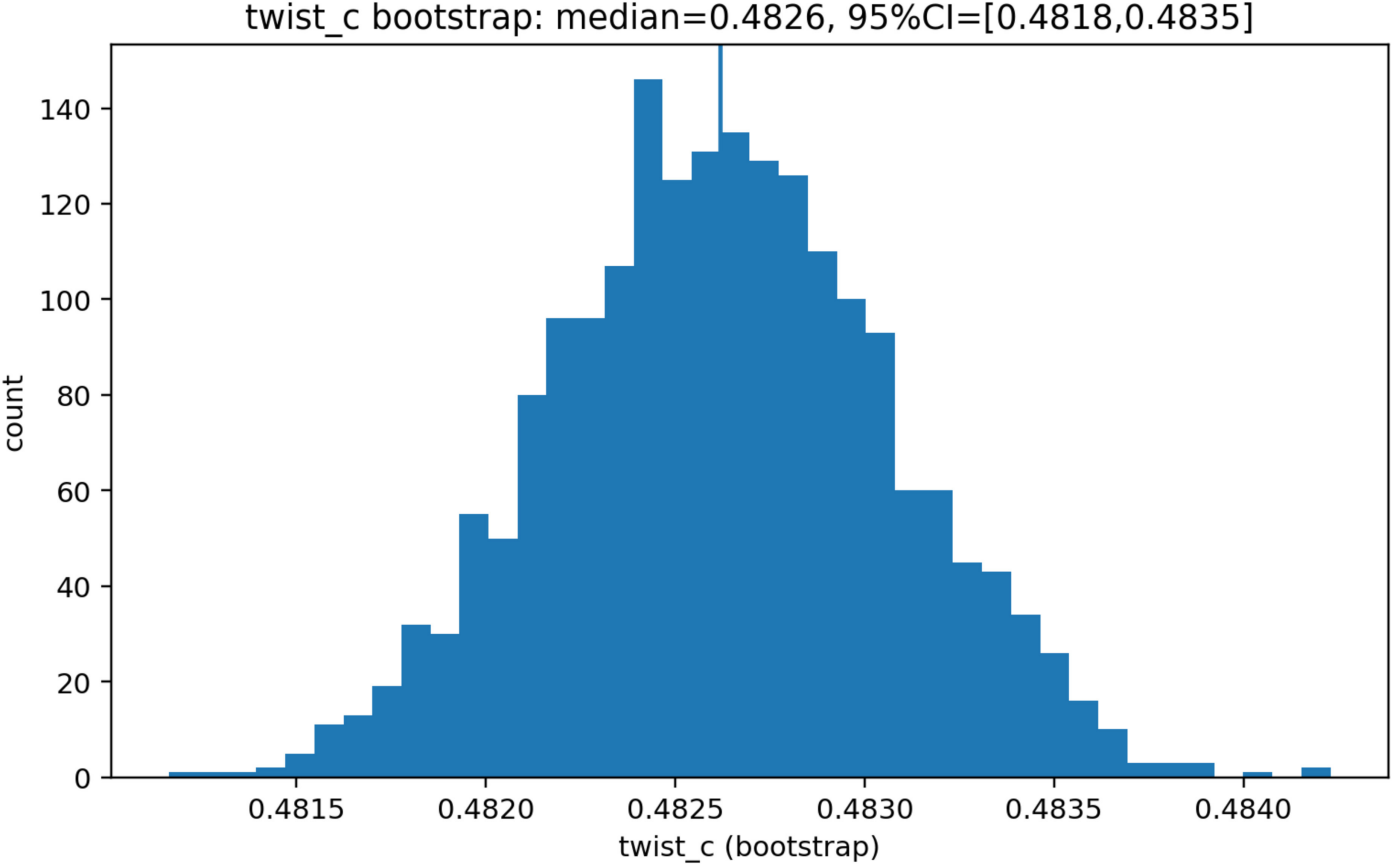
Bootstrap distribution of the transition point. Bootstrap distribution of the transition point θ_c estimated from resampled two-dimensional twist-scan data. The distribution is tightly concentrated around θ_c ≈ 0.483, with a narrow 95% confidence interval. These results indicate that the location of the ipsilateral-to-contralateral shift is highly stable and robust to resampling variability.

The estimated slope of the transition point as a function of delay was −0.000603931. Bootstrap analysis confirmed the robustness of this result (Figures 4, 5). The median transition point was 0.482618, with a 95% confidence interval of 0.481766–0.483507. The slope of the delay dependence had a median of −0.000612461, with a 95% confidence interval of −0.000777127 to −0.000447557.

These findings indicate that the location of the transition is governed primarily by the transformation parameter, whereas the effect of interhemispheric delay is comparatively small within the tested range.

### 3.6 Performance improves near and after the transition

We next examined how performance varies across the twist parameter (Figure 2). Mean objective values decreased from 10.634 at θ = 0.25 to 7.374 at θ = 0.45, reached their lowest values near the transition region (7.193 at θ = 0.50, 6.276 at θ = 0.55, and 6.246 at θ = 0.60), and then increased gradually at larger twist values.

These results show that the shift toward contralateral dominance in the model is associated with improved objective values, with the lowest values observed in the transition and immediate post-transition regime.

### 3.7 Summary of results

**FIGURE 6:**
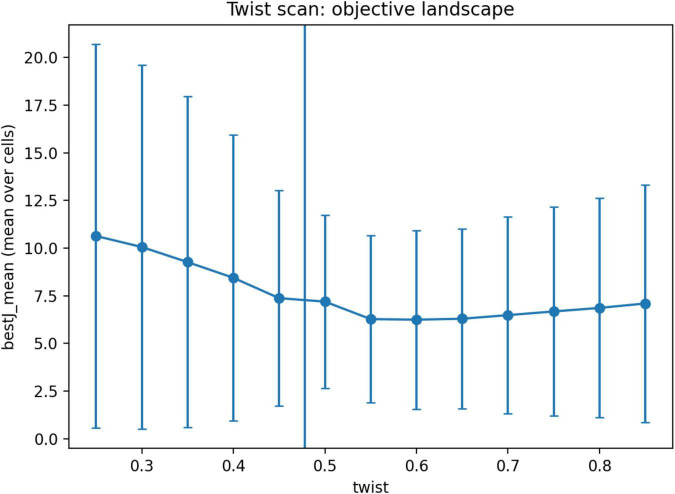
Objective landscape across the twist parameter. Mean objective value (bestJ_mean) as a function of the geometric twist parameter θ in the dense two-dimensional twist scan. Objective values decreased toward the transition region, reached their lowest values near and just above the estimated transition point (θ_c ≈ 0.483), and then increased gradually at larger θ values. These results indicate that the shift toward contralateral dominance occurs in a regime associated with lower objective values, rather than with increased cost.

Taken together, the results show a consistent pattern across analyses. In one-dimensional systems, contralateral architectures are dynamically viable but do not dominate and are sensitive to the structure of the objective function. In two-dimensional systems without geometric transformation, the optimal architecture is overwhelmingly ipsilateral, whereas under full inversion contralateral architectures dominate completely. Between these regimes, the system exhibits a sharp reorganization of the optimal architecture over a narrow range of the twist parameter (Figure 2), with a transition point located near θ_c ≈ 0.483. This transition is robust under bootstrap resampling (Figures 4, 5), weakly dependent on interhemispheric delay (Figure 3), and associated with lower objective values near and just above the transition region (Figure 6).

### 3.8 Mechanistic basis of the twist-dependent transition

**FIGURE 7:**
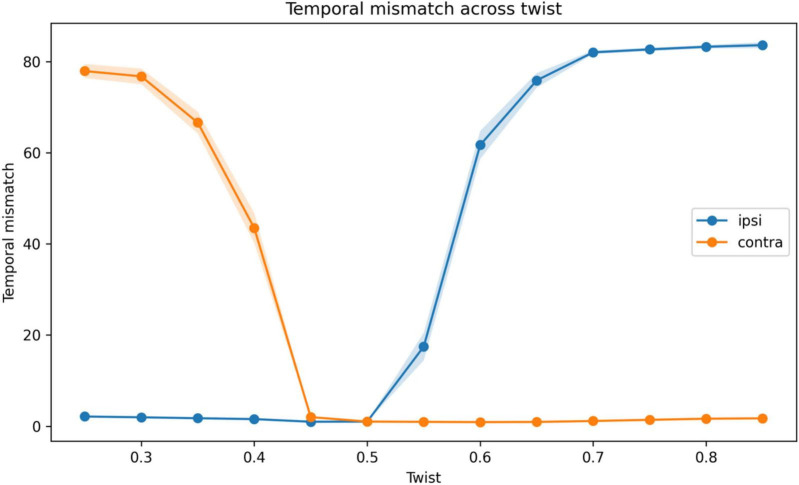
Temporal mismatch across the twist parameter. Mean temporal mismatch between required motor correction and produced motor output as a function of the twist parameter θ, shown for ipsilateral and contralateral architectures. Shaded regions indicate standard error across repetitions. At low θ, ipsilateral architectures exhibit lower temporal mismatch, whereas contralateral architectures show substantially higher mismatch. Near θ≈ 0.50, the difference between architectures becomes minimal, indicating a near-balanced regime. At higher θ, temporal mismatch increases sharply for ipsilateral architectures while remaining low for contralateral architectures, indicating improved temporal alignment under contralateral routing.

**FIGURE 8:**
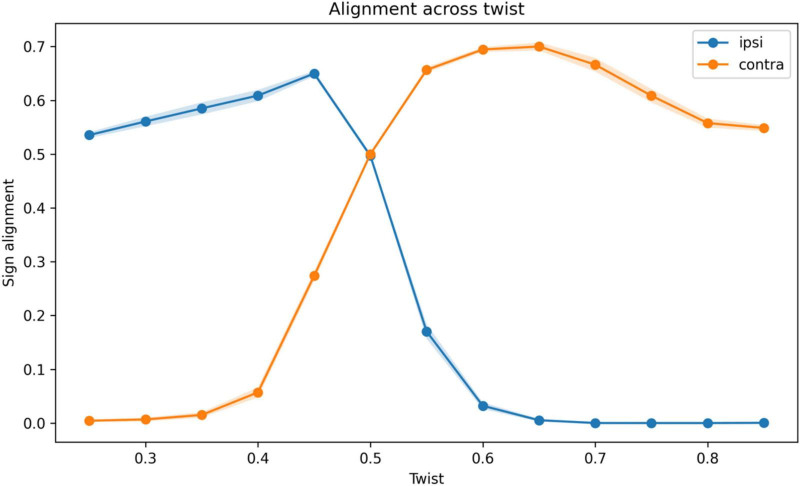
Alignment between required and produced motor signals. Mean sign alignment between required motor correction and produced motor output as a function of the twist parameter θ, shown for ipsilateral and contralateral architectures. Shaded regions indicate standard error across repetitions. At low θ, ipsilateral architectures exhibit higher alignment, whereas contralateral architectures show near-zero alignment. Around θ≈ 0.50, alignment values converge. At higher θ, contralateral architectures exhibit substantially higher alignment, while ipsilateral alignment decreases toward zero. This reversal indicates that contralateral routing improves the consistency between control signals and task demands under increased geometric transformation.

To directly test the mechanism underlying the reorganization of architecture, we quantified temporal mismatch and alignment between required motor correction and produced motor output across the twist parameter (Figures 7, 8).

Temporal mismatch showed a clear divergence between ipsilateral and contralateral architectures (Figure 7). At θ = 0.25, temporal mismatch was 2.1190 for the ipsilateral architecture and 77.9474 for the contralateral architecture. Near the transition region (θ = 0.50), the difference between architectures was minimal, with an ipsilateral-minus-contralateral difference of 0.0049. At higher twist values, temporal mismatch increased sharply for the ipsilateral architecture, while remaining low for the contralateral architecture.

A complementary pattern was observed in alignment (Figure 8). At θ = 0.85, sign alignment was 0.0005 in the ipsilateral architecture and 0.5487 in the contralateral architecture. Consistent with this, total tracking error strongly favored the contralateral architecture at high twist, with an ipsilateral-minus-contralateral difference of 8669.0757.

These results show that the shift in optimal architecture is associated with a reversal in temporal alignment. Under low twist, ipsilateral routing minimizes mismatch, whereas under high twist, contralateral routing provides superior alignment. Because the system includes interhemispheric competition, these differences are amplified into a rapid shift in the optimal architecture.

### 3.9 Sensitivity to objective weighting

To assess the dependence of the results on the specific weighting of the objective function, we performed a one-factor-at-a-time sensitivity analysis in which each objective weight was scaled to 0.5×, 1.0×, and 1.5× while the remaining weights were held fixed.

Across these perturbations, the qualitative structure of the results was preserved. In particular, the system consistently exhibited a transition from ipsilateral-dominated solutions at low twist to contralateral-dominated solutions at high twist.

The estimated transition point varied only modestly, ranging from *theta_c* = 0.4839 to 0.5023 across all tested weighting conditions. Contralateral dominance in the high-twist regime was also maintained, with the mean contralateral frequency for *theta* ≥ 0.70 ranging from 0.944 to 1.000 and the value at *theta* = 0.85 ranging from 0.963 to 1.000.

Sensitivity was greatest for the tracking term, particularly when its weight was reduced to 0.5×, but even in this condition the high-twist regime remained predominantly contralateral.

These results indicate that the existence of the ipsilateral-to-contralateral reorganization does not depend on a single specific choice of objective weights.

## 4 Discussion

The present study shows that contralateral routing can become functionally favored through the interaction between coordinate transformation, delayed signal propagation, and bilateral competition in a minimal sensorimotor system. The central result is not simply that contralateral architectures can outperform ipsilateral ones, but that the optimal architecture reorganizes sharply as a function of the transformation parameter. Within the present abstract control framework, this reorganization was robust across the tested parameter variations and was associated with lower objective values near and just above the transition region.

A central contribution of this work is the identification of a mechanistic basis for this reorganization. The additional analyses of temporal mismatch and sign alignment showed that the transition was associated with a reversal in temporal alignment between required motor corrections and produced motor outputs. Under low transformation, ipsilateral architectures minimized temporal mismatch and produced better-aligned control signals. As the degree of transformation increased, delayed processing in the ipsilateral pathway led to increasing misalignment. In contrast, contralateral routing maintained better alignment under these conditions, resulting in lower temporal mismatch and improved control performance. These findings indicate that the shift toward contralateral routing in the model is associated with improved temporal coordination in a delayed control system.

Importantly, this mechanism differs from explanations based solely on wiring economy or delay minimization. While wiring optimization has been proposed as an important organizing principle in neural systems (Cherniak, 1994; Bullmore and Sporns, 2012), the present results show that the transition was only weakly dependent on interhemispheric delay within the tested range. Instead, the dominant factor in the present framework was the interaction between coordinate transformation and temporal alignment. This suggests that routing preference cannot be understood fully in terms of connection length or transmission delay alone, but must also consider how signals are aligned with task-relevant coordinate frames.

This point allows the present results to be related more directly to classical neurobiological accounts of contralaterality. In visual systems, contralateral projections have long been discussed in relation to the preservation of orderly spatial relations under inversion of the sensory image, that is, in terms of maintaining coherent mappings of external space across sensory processing stages (Cajal, 1995; Jeffery, 2001; Kaas, 1997). The present model does not reproduce retinotopic anatomy or optical inversion directly. However, it is consistent with the more general idea that when sensory and motor coordinate systems are sufficiently inverted or mismatched, routing architecture becomes functionally consequential. Under this interpretation, the transformation parameter should be understood not as a literal anatomical or embryological twist, but as an abstract measure of the degree of transformation between sensory and motor coordinate systems. This interpretation is more consistent with the scope of the present model and avoids treating the parameter as a direct representation of a specific developmental event.

This interpretation also helps resolve the apparent tension between contralateral organization in some modalities and more ipsilateral organization in others. Contralateral organization is not uniform across sensory systems, and the present framework does not imply that all modalities should converge on contralateral routing. Rather, it predicts that contralateral routing becomes favored when the effective mapping between sensory and motor coordinates contains a strong topological inversion or mismatch. Systems such as vision, in which orderly spatial relations must be preserved across transformed coordinate frames (Cajal, 1995; Kaas, 1997), may therefore correspond to higher values of the transformation parameter. By contrast, systems such as olfaction, which are less strongly organized by a continuous topographic mapping of external space and are more ipsilateral in their primary projections, may correspond to lower-transformation regimes in which ipsilateral routing remains sufficient (Shepherd, 2004). In this sense, the present results do not propose a modality-independent rule that all neural systems should become contralateral, but rather a conditional dynamical principle whose relevance depends on the structure of the underlying coordinate transformation.

Another important aspect of the present findings is that the reorganization of architecture was not gradual. The system did not smoothly interpolate between ipsilateral and contralateral strategies. Instead, the optimal architecture shifted over a relatively narrow range of the transformation parameter. This behavior reflects the nonlinear interaction between delayed feedback and competitive selection. Near the transition region, relatively small differences in temporal alignment produced large differences in objective value, which were then amplified by interhemispheric competition. As a result, the system selected one dominant routing architecture rather than maintaining an even mixture of the two.

From a computational perspective, this work contributes to the broader question of how network architecture can be shaped by dynamical constraints. Previous studies have shown that delays, noise, and recurrent interactions can shape neural dynamics and functional organization (Deco et al., 2011; Breakspear, 2017). The present results extend this perspective by showing that transformations in effective sensorimotor mapping can reorganize the landscape of preferred bilateral architectures. In this sense, the study does not provide a direct anatomical account of vertebrate wiring. Rather, it identifies a minimal dynamical principle by which routing preference can shift when transformation, delay, and bilateral competition are jointly present.

An additional issue concerns the dependence of the results on the specific weighting of the objective function. Because the objective combines multiple cost components, one possible concern is that the observed transition might reflect a particular weighting choice rather than a more general property of the system. To address this, we performed a one-factor-at-a-time sensitivity analysis in which each objective weight was scaled to 0.5×, 1.0×, and 1.5× while the remaining weights were held fixed. Across these perturbations, the qualitative structure of the results was preserved. The estimated transition point varied only modestly, and contralateral dominance in the high-transformation regime was maintained across tested weighting conditions. Sensitivity was greatest for the tracking term, but even in this case the high-transformation regime remained predominantly contralateral. These results indicate that the existence of the ipsilateral-to-contralateral reorganization does not depend on a single specific choice of objective weights.

Several limitations should be noted. First, the model is intentionally minimal, and the left and right pathways are abstract processing channels rather than anatomically embedded hemispheres with explicit spatial positions, cell types, or developmental identities. Second, the architecture space is discrete and simplified, and does not capture the full diversity of biological neural networks or the partial decussation often observed in real systems (Jeffery, 2001). Third, motor output was fixed in the present framework in order to isolate the effect of sensory laterality and interhemispheric coupling, and the model therefore does not capture the full range of biological sensorimotor crossing patterns. Fourth, although the qualitative results were preserved under moderate perturbations of objective weighting, the precise location of the transition point still depends to some extent on how performance is operationalized. Fifth, the study is theoretical and does not incorporate empirical neural or developmental data directly. These limitations constrain the strength of biological claims that can be made from the present framework.

These limitations suggest several directions for future work. One important extension is to embed sensory, integrative, and motor elements within an explicit shared spatial framework, for example by assigning positions, distances, and connectivity constraints in two- or three-dimensional space. Such an extension would allow laterality to be evaluated under physically instantiated geometry rather than abstract channel assignment alone. It would also be valuable to test whether the same mechanism is preserved in more realistic recurrent or heterogeneous network architectures, and to examine how routing preference depends on modality-specific task structure. In addition, future work could relate the present framework more directly to empirical systems by asking whether modalities with stronger effective coordinate inversion exhibit stronger tendencies toward contralateral routing. Finally, analytical treatment may help clarify why the transition occurs near a specific value of the transformation parameter and how that threshold depends on the balance between alignment, delay, and competitive interactions.

In summary, the present study identifies a minimal dynamical mechanism by which contralateral routing can become functionally favored in delayed bilateral control systems when sensory and motor coordinate systems are sufficiently transformed. By showing that contralateral architectures can improve temporal alignment under high transformation and that this advantage is amplified through bilateral competition, the results provide an abstract theoretical link between classical geometric accounts of contralaterality and systems-level dynamical considerations.

## Data Availability

The datasets presented in this study can be found in online repositories. The names of the repository/repositories and accession number(s) can be found below: https://doi.org/10.5281/zenodo.18885314.
